# Pseudomyxoma peritonei of appendiceal mucinous neoplasm origin: A case report and review of literature

**DOI:** 10.1016/j.radcr.2024.08.158

**Published:** 2024-09-28

**Authors:** Ahmed Hafez Mousa, Houriah Yasir Nukaly, Rayyan Rafat Samman, Samratul Fuadah, Bushra Wadi Bin Saddiq, Shahad Jamal Alshowaikhat, Islam Khalid

**Affiliations:** a Department of Neurosurgery, Graduate Medical Education Department (GME), Mohammed Bin Rashid University of Medicine and Health Sciences (MBRU), Dubai Health, Dubai, United Arab Emirates; b College of Medicine and Surgery, Batterjee Medical College, Jeddah, Saudi Arabia; c Department of Surgery, Faculty of Medicine, Suez Canal University Hospitals, Ismailia, Egypt

**Keywords:** Appendiceal mucinous neoplasm, Appendix cancer, Pseudomyxoma peritonei, Mucinous adenocarcinoma, Case report

## Abstract

Appendiceal mucinous neoplasms, a rarity comprising less than 1% of all cancers, present intricate challenges in clinical management, and their incidence is on the rise. Notably, these neoplasms tend to metastasize intraperitoneally, leading to peritoneal carcinomatosis and concurrent accumulation of mucinous material, resulting in pseudomyxoma peritonei. Due to its spectrum of presentation, the classification of the appendiceal mucinous neoplasms remains a controversial subject with a range of management from a simple appendicectomy to a complex hyperthermic intraperitoneal chemotherapy (HIPEC). A 42-year-old Chadian male presented to the hospital with a sudden onset of right lower abdominal pain radiating to the inguinal region for 24 hours, associated with nausea and vomiting. The abdomen was distended and ascitic. Laboratory investigations revealed anemia, leukocytosis, hypernatremia, hypokalemia, elevated ESR, high CEA marker, and normal CA19-9. An abdominopelvic CT with contrast demonstrated extensive ascites and cystic masses in the liver, and pancreas with soft tissue thickening of the cecum; however, the appendix is not well-delineated. Patient was managed with chemotherapy and HIPEC followed by removal of all the affected parts. Nodules of the peritoneum and liver were submitted for histopathological analysis and a final diagnosis of pseudomyxoma peritonei of primary appendicular origin was established. This case highlights a case of extensive pseudomyxoma peritonei of appendicular origin managed aggressively by HIPEC and multiple resections of the involved organs. Prognosis of such a case is determined by the grade of the appendiceal tumor and the extent of invasion.

## Introduction

Appendiceal Mucinous Neoplasm (AMN) is a rare type of appendiceal neoplasm originating from the appendiceal epithelium and accounting for less than 1% of gastrointestinal tumors. It has been reported that the incidence continues to increase at a rate of 3.1% per year, along with a decline in the age of diagnosis [1]. Amongst tumors arising from the appendix, AMN has been found in approximately 0.2%-0.3% of histopathological samples [2]. Due to its indolent course, diagnosis can be incidental due to its nonspecific manifestations in earlier stages; however, more commonly, a manifestation similar to acute appendicitis and with concurrent abdominal pain and distention, can lead to a suspicion of intraperitoneal spread of tumor [3,4]. Pseudomyxoma peritonei (PMP) is also an uncommon occurrence with an estimated incidence of 1-2 per million per year [2]. It is characterized by diffuse intra-abdominal gelatinous collections with mucinous implants which is commonly due to the rupture of mucinous tumors of which the most common primary origin is AMN followed by ovarian tumors [5,6].

As of recent, the diagnosis and management of appendiceal tumors have been subjects to controversial discourse. However, the most recent consensus on the guidelines for appendiceal tumors anointed by the Peritoneal Surface Oncology Group International (PSOGI) and the eighth Edition of the American Joint Committee on Cancer Staging (AJCC), provides a clearer understanding of the characterization thereby reflecting a more accurate histopathological diagnosis and treatment [7]. The management and prognosis of MAN with or without concurrent PMP is highly influenced by the grading and staging of neoplastic tissue. It ranges from a simpler hemicolectomy with appendectomy for lower grade tumors to the more aggressive cytoreductive surgery (CRS) and Hyperthermic Intraperitoneal Chemotherapy (HIPEC) for coexisting PMP which has shown to considerably improve survival [8]. We report a case of a 42-year-old patient who presented with right lower abdominal pain for 24 hours and obvious abdominal distention. The patient was diagnosed with pseudomyxoma peritonei of primary mucinous appendiceal neoplasm origin and treated with CRS and HIPEC.

## Case presentation

A 42-year-old Chadian male patient was referred to our hospital as a case of metastatic mucinous adenocarcinoma of the appendix discovered on CT scan. He presented with an acute onset of right lower abdominal pain of 24 hours of evolution, 9/10 in severity, radiating to the inguinal region associated with nausea and vomiting. The patient denied having fever, chills, weight loss nor any other constitutional symptoms. He is medically free. Past medical and family history were unremarkable, and the rest of his history was noncontributary.

Upon physical examination, the patient's vitals were as follows: blood pressure: 120/70 mmHg, temperature: 37OC, heart rate: 120 bpm, respiratory rate: 26 breaths per minute, SPO_2_ 96%, and body mass index (BMI) of 22.41 kg/m2. The patient is alert, and well-oriented to place, time and person, with average built, looks in agony, and lying in bed flexing his legs towards the abdomen. Remarkably, there is a distinct abdominal distention, and involuntary guarding with clear evidence of peritoneal irritation, but there are no signs of swellings, masses, nor hyperesthesia. Also, there is rebound tenderness, and positive McBurney's sign. There are no palpable lymph nodes, abdominal organs or masses. The intestinal sounds were audible with no vascular sounds. The rest of the examination was unremarkable. A series of laboratory and radiological investigations were ordered. All parameters of complete blood count (CBC) were within normal limits except for a low hemoglobin level of 11.3 g/dL, and an elevated white blood cell (WBC) (16.20*109/L), segmented neutrophil was also high (59.80%). erythrocyte sedimentation rate (ESR) (53 mm/hour) was found to be markedly elevated. The patient's blood group is O+ and negative for antibodies. Basic metabolic panel and venous blood gas (VBG) were also done. The results showed a low PH level of 7.29, low PCO2 level of 26 mmHg, low HCO3 level of 12 mEq/L and high lactate level of 5.2 mmol/L. Moreover, the results indicated high sodium (Na+) level of 149mmol/L and low potassium (K+) level of 2.8 (mEq/L). Additionally, serum creatinine (CRE2) was marginally low (0.64 mg/dL). The initial serum carcinoembryonic antigen (C.E.A) was high (76.21 ng/mL). However, serum cancer antigen 19-9 (CA 19-9) is found to be normal (<2.06 U/mL). Table 2 provides an overview of the abnormal results. Lastly, coagulation profile was within normal limits and anti-HCV, HBs Ag, and HIV Ag/Ab, were found to be nonreactive.

**Fig. 1:**
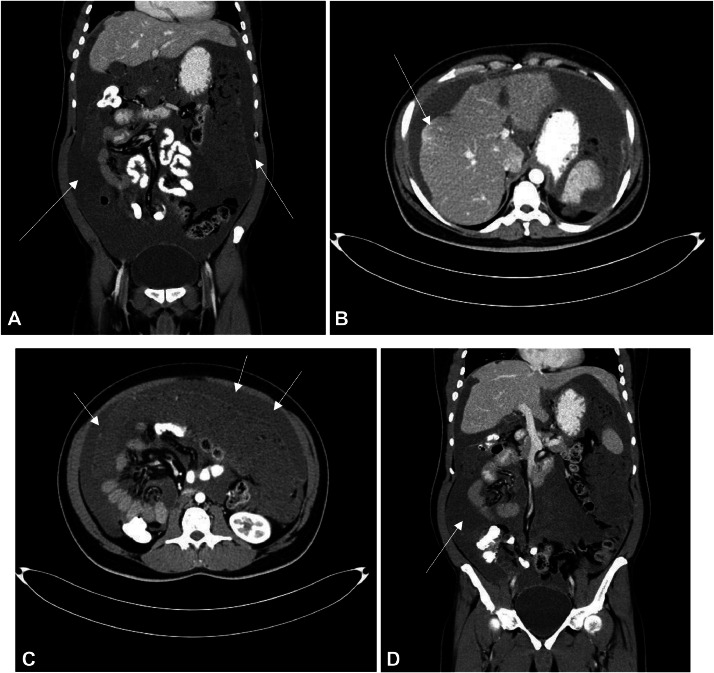
Abdominopelvic CT scan with contrast denoting: (A) Extensive ascites on coronal section (B) axial section at the level of the liver showing small indentation of the hepatic surface by the fluid collection impressive of mucinous content (C) Axial section postcontrast at the level of kidneys showed extensive omental thickening. (D) Coronal section postcontrast demonstrating soft tissue thickening seen related to the cecum, otherwise, the appendix is not well-delineated.

An abdominopelvic computed tomography (CT scan) with contrast and oral and iv and rectal enema (Fig. 1); revealed a de novo cystic mass lesion seen in the left hypochondrium indenting the left hepatic lobe measuring 4 × 3.5 cm (Fig. 1B), in the previous study there was mild indentation. There is another small cystic lesion seen indenting the pancreatic tail measuring 1 cm (Fig. 1B). Progression of the right sub hepatic peritoneal lesion with de novo mild indentation of the segment VI of the right hepatic lobe (Fig. 1B). The rest of the peritoneal soft tissue and fluid are essentially stationary (Fig. 1A). There is free flow of the orally and rectally administrated contrast with no bowel masses or mural thickening in the remaining bowel loops (Fig. 1A).

Later, the patient was scheduled for U/S guided aspiration and pig tail insertion with cytology. Aspiration of left hypochondrium cyst was done and around 40 mL of turbid yellowish fluid drained with no immediate postprocedure complications. Besides, paracentesis was also done and ascitic fluid report revealed 60 mL of reddish turbid fluid (4 smears and cell blocks were prepared). US guided omental tissue biopsy from right lower quadrant of the abdomen showed multiple fragments of tan tissue measured 0.5 × 0.5 × 0.1 cm on gross examination. Omental biopsy and immunohistochemistry report strips of atypical glandular epithelial cells with mucin extravasation, consistent with low grade peritoneal mucinous neoplasia, target cells are positive for MOC31 and negative for calretinin. The majority of these tumors arise from appendiceal mucinous neoplasm and the diagnosis of pseudomyxoma peritonii of primary appendicular origin was established.

He was managed with 3 cycles of chemotherapy and the patient underwent hyperthermic intraperitoneal chemotherapy (HIPEC) with extensive pelvic and abdominal peritonectomy, subtotal colectomy, splenectomy, subtotal gastrectomy, porta hepatis lymphadenectomy, appendectomy with ileostomy. Intraoperatively, R0 resection could not be obtained due to aggressive peritoneal disease. Excision of peritoneal nodules, resection of liver nodules, as well as porta hepatic nodule resection, and finally, doughnuts of anastomosis were all sent to the histopathologist in 4 containers. No surgical complications were encountered during the procedure.

Histopathology examination reported the following: grossly, excised peritoneal nodules; multiple fibrofatty tissue measured 10 × 5 × 1 cm, with cut sections showed multiple gelatinous mucoid whitish nodules. Resected liver nodules; liver tissue measured 2 × 1.5 × 1 cm, with cut sections showed multiple gelatinous mucoid whitish nodules. Resected porta hepatic nodule; soft tissue fragment measured 7 × 3.5 × 1 cm, with cut sections showed gelatinous mucoid whitish nodules. Doughnuts of anastomosis; 2 soft tissue fragments measured 2.5 × 2 cm, and 3 × 0.5 cm. Microscopically, all of the excised peritoneal nodules, resected liver nodules, and resected porta hepatic nodule showed: metastatic mucinous adenocarcinoma of the appendix. Doughnuts of anastomosis revealed the presence of foci of acellular mucin deposits. The ileal mucosa was negative for invasive carcinoma.

Of note, 4 months later, the patient developed high liver enzymes (SGPT 162 U/L) and the chemotherapy session was postponed. The patient underwent abdominopelvic ultrasound which displayed minimal thin rim of inter-bowel free fluid seen around the bowel loops that shows subtle mucosal mural thickening. A diagnosis of colorectal cancer was made and he was managed with HIPEC, ileocolic anastomosis and partial liver metastasis resection. The patient was admitted to the intensive care unit (ICU) for 3 days, after which he was transferred to the ward. On the fifth day post-HIPEC, the patient was advanced to soft diet and received 1 unit of packed red blood cells (PRBCS). The patient was advised to mobilize more and was discharged with drain and had an overall good recovery.

**Table 1:** Literature review table.

Study	Age (Y)	Gender	Clinical Presentation	Laboratory Tests	Diagnostic Modalities	Diagnostic findings	Association with other diseases	Management	Recurrence
Kusuyama et al. [9]	25	F	right lower abdominal pain	CEA, HCG, CA15-3, CA19-9, CA125 and SCC antigen are all normal Immunological fecal occult blood reaction (RHPA) was negative	a. Barium enema b. Colon fiberscopy c. Abdominal CT d. Abdominal US	a. good wall distensibility in the ileocecum and no abnormal signs b. small red elevation deep in the cecum contralatereal to Bauhin's valve c. no masses around ileocecum, normal uterus and ovaries and no ascites d. small amount of ascites in Douglas' pouch	multicystic peritoneal mesothelioma	Laporotomy Left oopherectomy, omentectomy, partial cecectomy and partial ilectomy (with resection of the Meckel's diverticulum) were performed Cis-diamminedichloroplatinum (CDDP) 100mg was given by intraperitoneal injection and a catheter was placed in Douglas' pouch	No
Konstantinos et al. [10]	64	F	hypogastric and right lower quadrant abdominal pain for 24 hours	normal	a. Pelvic US b. Pelvic CT	a. small quantity of free liquid located in the Douglas area and the right parametrium b. normal internal organs (uterus and ovaries)	first operation: appendicectomy and thorough cleaning of the peritoneal cavity second operation: right hemicolectomy	open laparotomy on a Mac Burney section Appendicectomy and cleaning of the peritoneal cavity	N/A
Noh et al. [11]	72	F	dyspepsia and weight loss	normal	a. pelvic CT	a.mesoappendiceal mass-like lesion with calcifications orginating from right lower quadrant large volume of ascites with omental thickening	N/A	appendectomy with palliative currettage	No
Suh et al. [12]	73	F	worsening indigestion and abdominal distension	CA125 = 51.79 U/mL Human epididymis protein 4 = 98.07 ng/mL	a. Pelvic US b. MRI	a. large multiseptated cystic mass on the right ovary with a large amount of peritoneal fluid in the upper abdomen b. large multilocular cystic mass, measuring 21.8 cm. No mural nodules or enhancing solid components were observed within the mass; however, ascites and mild scalloping of the liver surface were evident. hypointense septa in the fluid collection at the cul-de-sac.	ovarian tumor	Laporotomy, bilateral salpingo-oophorectomy, hysterectomy, ileocecectomy, omentectomy, excisions of multifocal peritoneal mucinous implants and peritoneal lavage were performed	No
Watanabe et al. [13]	68	F	anorexia, abdominal distension, abdominal pain in the lower-right abdomen	WBC = 13,600/μL CRP = 33.8 mg/dL Serum albumin level = 3.4 g/dL Slight renal dysfunction (BUN 108 mg/dL, Creatinine 4.25 mg/dL) CEA = 37 ng/mL CA19-9 = 113 U/mL CA125 = 124 U/mL	a. abdominal CT b. MRI c. aspiration of ascites	a. massive ascites and cystic mass in the lower right abdomen that ruptured to the abdominal cavity b. cystic tumor was arising from appendix c. yellow and cloudy	right-sided inguinal hernia and uterine prolapse	laporotomy, appendectomy and bilateral oopherectomy and irrigation of abdominal cavity using 3000ml of dextran solution fixation of pelvic diaphragm by sutures and repair of the inguinal hernia via another incision after 2 months, administration of S1 was done to prevent relapse	No
Ning et al. [14]	70	M	abdominal pain and distension for 1 month	WBC: 9.02 × 10^9/L NET%: 78.90% CEA: >60.00 μg/L	a. abdominal CT	a. peritoneal effusion and bowel dilatation	rectal carcinoma	emergency exploratory laparotomy cytoreductive surgery, enterolysis, intestinal decompression and special tumor treatment CC1 cytoreduction, radical resection of rectal carcinoma	No
Ayadi et al. [15]	62	F	abdominal pain	ACE = 138.08 ng / mL CA 125 = 306.60 U/mL CA 19-9 = 527.85 U/mL	a. CT b. MRI	a. curvilinear mural calcifications pelvic cystic mass nodular thickening of the peritoneal reflections with stranding thickening of the omentum associated with scalloping of the liver surface b. cystic mass attached to caecum with a discontinuous wall with curvilinear calcifications no abnormalities with the ovaries	N/A	appendix removal was not possible due to presence of multiple adhesions and carcinomatosis nodules treatment was only with neoadjuvant folfox-based chemotherapy	No
Geisel et al. [16]	58	M	N/A	normal	a. Intraoperative diagnosis b. MRI	a. mucocele of appendix b. mucinous implants in all quadrants of the abdomen	N/A	oral administration of bromelain and acetylcysteine	-
Gupta et al. [17]	64	M	abdominal pain	N/A	a. CT b. colonoscopy	a. 51.96mm mass in the right iliac fossa obstructing the view of the appendix with a focus of calcification infiltration into the adjacent fat and abnormal soft tissue thickening of the peritoneal reflection along the right paracolic gutter multiple peritoneal nodules in the upper abdomen b. no abnormalities in the mouth of the appendix and the caecum	incidental grade 1 well-differentiated neuroendocrine tumor in the LAMN	cytoreductive surgery with right hemicolectomy and cholecystectomy and hyperthermic intraperitoneal chemotherapy (HIPEC)	N/A
Rong et al. [18]	43	F	intermittent fever and abdominal distension for 1 year	CA125 = 63.76 U/mL CA199 = 44.2 U/mL	a. gynecological US b. plain MRI c. exploratory laporoscopy	a. pelvic effusion with flocculent echo b. massive ascites and thickening of the omentum majus and mesentery c. yellowish ascites was seen in the pelvic cavity, and the surfaces of intestines, peritoneum, vesicoper- itoneum, greater omentum, bilateral adnexa, uterorectal fossa, and liver were covered with flocculent material, and the appendix was enlarged and thickened by 6*6 cm	N/A	exploratory laporoscopy with removal of flocculent material and excision of the appendix	N/A

**Table 2:** Abnormal lab results.

Test	Normal values	Result
Complete Blood Count (CBC) with differentials		
Hemoglobin (HGB)	13-17	11.3 g/dL
White Blood Cells (WBC)	4-10	16.20*109/L
Segmented Neutrophil		59.80%
Erythrocyte Sedimentation Rate (ESR)	4-10	53 mm/hour
Basic Metabolic Panel (BMP)		
Serum Creatinine (CRE2)	0.72-1.25	0.64 mg/dL
Na^+^	136-145 mmol	149 mmol/L
K^+^	3.6 to 5.2 mEq/L	2.8 mEq/L
Venous Blood Gast (VBG)		
pH	7.31-7.41	7.29
pCO_2_	41-51 mmHg	26 mmHg
HCO_3_	23-29 mmHg	12 mEq/L
Lactate	< 1.0 mmol/L	5.2 mmol/L
Tumor Markers		
C.E.A	≤ 3 ng/mL	76.21 ng/mL

## Discussion

Appendiceal lesions are infrequent; in a 10-year Dutch research, the frequency of appendiceal lesions was 9 per million people per year, with one in every 113 removed appendices having a lesion (benign or malignant in nature) [2]. Although more recent studies show a greater disease frequency, malignant lesion rate, and younger age at diagnosis, the benign-to-malignant ratio was 3 to one. The Peritoneal Surface Oncology Group has established an agreement on categorization of mucinous appendiceal neoplasms, classifying them as adenoma, low‐grade appendiceal mucinous neoplasms (LAMNs), high-grade appendiceal mucinous neoplasms (HAMNs), and mucinous adenocarcinomas, which has been a source of contention for many years [19]. High-grade adenocarcinomas are cytologically malignant and have infiltrative invasion, lymph node metastasis, and behavior comparable to extra-appendiceal mucinous adenocarcinomas. At the opposite end, mucinous neoplasms localized to the mucosa tend to be benign. Despite the absence of malignant histologic characteristics, certain cases in the middle have the potential to metastasize inside the abdomen. They are "diverticulum-like," pushing invasion of low-grade epithelium through the appendix with or without associated intra-abdominal mucin organization. The latter disease is known colloquially as "pseudomyxoma peritonei" [20]. Tumor markers of appendiceal mucinous adenocarcinoma can be elevated, such as Serum cancer antigen 19-9 (CA199) and carcinoembryonic antigen (CEA). Diagnosis can be confirmed by pathological examination. An MRI would reveal the metabolic state of the lesion with the anatomical structure, which is helpful for improving the detection rate of the tumor and guiding the development of the diagnosis and treatment plan [21].

Several case reports revolving around similar conditions to the one mentioned in this case report have been referenced to state the clear similarities and differences found. The following references were used, 1. Zou *et al. [*22], 2. Makino *et al. [*23], 3. Wu *et al. [*24], 4. Konnai *et al. [*25], 5. Amir *et al. [*26], 6. Chen *et al. [*27]. Our patient was a 42-year-old male, presenting at a younger age than most cases with similar conditions, meanwhile the ages and genders of the previously mentioned cases were as follows, 64-year-old female [22], 62-year-old male [23], 60-year-old male [24], 52-year-old female [25], 62-year-old female [26], and 70-year-old female [27]. Only 1 case shared the chief complaint of acute right lower abdominal pain [23], one complained of lower back pain for 1 year [22], one complained of abdominal distention spanning 4 years [24], one complained of excessive flow and duration of her menstrual cycle [25], one complained of acute right flank pain [26], and the final one complained of a palpable pelvic mass that was detected 2 days ago [27]. All 6 cases, plus our own, were found to have a lesion located in the lower right abdomen, while 2 of them had metastatic lesions found on the ipsilateral kidney [22] and retroperitoneum [27]. The radiological modality for diagnosis of the disease was a CT scan which applied to all the cases, except for one that was diagnosed through transvaginal ultrasound and MRI [25]. The radiological findings in our case were a de novo cystic mass lesion seen in the left hypochondrium indenting the left hepatic lobe measuring 4 × 3.5 cm, another small cystic lesion seen indenting the pancreatic tail measuring 1 cm, and Progression of the right sub hepatic peritoneal lesion with de novo mild indentation of the segment VI of the right hepatic lobe. In the first mentioned case, the findings were a right kidney enlargement, multiple hyperdense nodules measuring approximately 2.3 × 1 cm in the right renal calyces, and right ureteral wall thickening. The appendix was thickened with mild enhancement, measuring 1.4 cm in diameter with a hypodense lumen [22]. The second case showed an appendiceal enlargement to a maximum diameter of 4.5 cm and the lumen had expanded to 3.2 cm with homogeneous fluid stored inside [23]. The third case showed a mass containing solid and cystic components arising from the appendix [24]. The fourth case showed a cystic lesion near the right iliopsoas muscle, showing high intensity on T2-weighted imaging and low intensity on T1-weighted imaging [25]. The fifth case showed an appendiceal enlargement measuring 7 cm in length, with marked dilation close to the distal margin measuring at 5.8 cm in diameter and a 2.4 cm simple cyst was detected in the right kidney [26]. While the final case showed a mass composing of mixed, mainly cystic heterogeneous mass, measuring 6.5 × 4.2 cm at the right end and increased echogenicity in the center measuring 4.5 × 2.1 cm [27]. Laboratory analysis for carcinoembryonic antigen (CEA) was found to be high in our case at 76.21 ng/mL and at 25.6 ng/mL in case 1 [22]. Cancer antigen (CA 19-9) was found to be high in case 6 at 64.4 IU/mL (reference range: 0-39 IU/mL) [27]. The treatment strategy followed for our case was 3 cycles of chemotherapy and hyperthermic intraperitoneal chemotherapy with extensive pelvic and abdominal peritonectomy, subtotal colectomy, splenectomy, subtotal gastrectomy, porta hepatis lymphadenectomy, appendectomy with ileostomy, excision of peritoneal, liver and porta hepatic nodules. In the first case, the management plan consisted of an open appendectomy plus abdominal exploration under general anesthesia, followed by a laparoscopic right nephrectomy 2 months later [22]. The second case was managed by performing a laparoscopic dissection of the cecum, ileocecal resection and anastomosis, and lymphadenectomy [23]. The third case was managed through exploratory laparotomy, followed by cytoreductive surgery and hyperthermic intraperitoneal chemotherapy 1 month later [24]. The fourth case had a history of bilateral mastectomy at the age of 51, with a history of endometrial hyperplasia, so she was managed by a total hysterectomy and laparoscopic risk-reducing salpingo-oophorectomy, in addition to a laparoscopic partial cecal resection and anastomosis [25]. The fifth case was managed through elective laparoscopic appendectomy [26]. The final case being complicated by retroperitoneal extension was managed by a laparoscopic exploratory surgery consisting of the resection of the appendiceal and splenic lesions, plus administration of hyperthermic intraperitoneal chemotherapy [27]. In all cases, postoperative analysis of the appendix and its contents indicated features of appendiceal mucinous neoplasm.

As an outcome, the prognosis of appendix mucinous adenocarcinoma is mostly determined by whether the tumor is at an advanced stage, the degree of malignancy, and the presence of a peritoneal pseudomyxoma. The expansion of mucus beyond the right lower quadrant is a standalone factor influencing the disease's bad prognosis. The presence of peritoneal pseudomyxoma and symptoms of probable infiltration surrounding the abdominal organs point to a dismal prognosis. If the patient has minor clinical symptoms and no evident infiltration or spread of surrounding tissue, removing the appendix tissue completely can increase the patient's survival time [21].

## Conclusion

In conclusion, appendiceal mucinous adenocarcinomas, although rare, carry a significant prognosis determined by factors like tumor stage, malignancy degree, and the presence of peritoneal pseudomyxoma. Our case underscored a comprehensive treatment strategy involving 3 cycles of chemotherapy and hyperthermic intraperitoneal chemotherapy. Extensive surgical interventions, including pelvic and abdominal peritonectomy, subtotal colectomy, splenectomy, subtotal gastrectomy, porta hepatis lymphadenectomy, appendectomy with ileostomy, and excision of peritoneal, liver, and porta hepatic nodes, were crucial in managing this condition. This emphasizes the critical importance of early detection of mucinous adenocarcinoma to prevent its involvement with nearby major organs.

## Photo consent statement

Written consent for the use of photographs in this manuscript has been obtained from the individuals depicted in the images. The individuals have been assured of their anonymity and understand that these images will be used exclusively for educational and illustrative purposes within this manuscript.

## Author contribution

**Ahmed Hafez Mousa:** Conceptualization; data curation; writing—original draft. **Houriah Yasir Nukaly:** Data curation; methodology; writing—original draft. **Rayyan Rafat Samman:** Methodology; writing—original draft, review and editing, publication. **Samratul Fuadah:** Methodology; writing—original draft, review and editing. **Bushra Wadi Bin Saddiq:** Methodology; writing—original draft. **Shahad Jamal Alshowaikhat:** Data curation; writing—original draft. **Islam Khalid:** Conceptualization; project administration; supervision; writing—original draft; review and editing.

## Patient consent

Informed consent was obtained from the patient and/or legal guardian for the publication of this case report. The patient and/or legal guardian were informed that their identity would be kept confidential, and all personal identifying information would be removed to ensure anonymity. They were also made aware that the case report may be published in medical literature or presented at medical conferences. They were informed that their participation was voluntary and that they could withdraw their consent at any time.
